# The tunable pReX expression vector enables optimizing the T7-based production of membrane and secretory proteins in *E. coli*

**DOI:** 10.1186/s12934-017-0840-4

**Published:** 2017-12-16

**Authors:** Grietje Kuipers, Alexandros Karyolaimos, Zhe Zhang, Nurzian Ismail, Gianluca Trinco, David Vikström, Dirk Jan Slotboom, Jan-Willem de Gier

**Affiliations:** 1 Department of Biochemistry and Biophysics, Center for Biomembrane Research, Stockholm University, SE-106 91 Stockholm, Sweden; 2 Xbrane Biopharma AB, SE-111 45 Stockholm, Sweden; 3 University of Groningen, Groningen Biomolecular Sciences and Biotechnology Institute, NL-9747 AG, Groningen, The Netherlands

**Keywords:** *Escherichia coli*, Protein production, Membrane protein, Secretory protein, T7 RNA polymerase, Lemo21(DE3)

## Abstract

**Background:**

To optimize the production of membrane and secretory proteins in *Escherichia coli,* it is critical to harmonize the expression rates of the genes encoding these proteins with the capacity of their biogenesis machineries. Therefore, we engineered the Lemo21(DE3) strain, which is derived from the T7 RNA polymerase-based BL21(DE3) protein production strain. In Lemo21(DE3), the T7 RNA polymerase activity can be modulated by the controlled co-production of its natural inhibitor T7 lysozyme. This setup enables to precisely tune target gene expression rates in Lemo21(DE3). The *t7lys* gene is expressed from the pLemo plasmid using the titratable rhamnose promoter. A disadvantage of the Lemo21(DE3) setup is that the system is based on two plasmids, a T7 expression vector and pLemo. The aim of this study was to simplify the Lemo21(DE3) setup by incorporating the key elements of pLemo in a standard T7-based expression vector.

**Results:**

By incorporating the gene encoding the T7 lysozyme under control of the rhamnose promoter in a standard T7-based expression vector, pReX was created (ReX stands for Regulated gene eXpression). For two model membrane proteins and a model secretory protein we show that the optimized production yields obtained with the pReX expression vector in BL21(DE3) are similar to the ones obtained with Lemo21(DE3) using a standard T7 expression vector. For another secretory protein, a *c*-type cytochrome, we show that pReX, in contrast to Lemo21(DE3), enables the use of a helper plasmid that is required for the maturation and hence the production of this heme *c* protein.

**Conclusions:**

Here, we created pReX, a T7-based expression vector that contains the gene encoding the T7 lysozyme under control of the rhamnose promoter. pReX enables regulated T7-based target gene expression using only one plasmid. We show that with pReX the production of membrane and secretory proteins can be readily optimized. Importantly, pReX facilitates the use of helper plasmids. Furthermore, the use of pReX is not restricted to BL21(DE3), but it can in principle be used in any T7 RNAP-based strain. Thus, pReX is a versatile alternative to Lemo21(DE3).

**Electronic supplementary material:**

The online version of this article (10.1186/s12934-017-0840-4) contains supplementary material, which is available to authorized users.

## Background

The *Escherichia coli* T7 RNA polymerase (T7 RNAP)-based protein production strain BL21(DE3) in combination with T7 promoter-based expression vectors is widely used to produce recombinant proteins [1, 2]. In BL21(DE3), expression of the gene encoding the target protein is transcribed by the chromosomally encoded T7 RNAP, which transcribes eight times faster than *E. coli* RNAP [3–5]. The gene encoding the T7 RNAP is under the control of the *lac*UV5 promoter (P*lac*UV5), which is a strong variant of the wild-type *lac* promoter [6, 7]. Addition of isopropyl-β-d-1-thiogalactopyranoside (IPTG) leads to expression of the gene encoding the T7 RNAP. The T7 RNAP specifically recognizes the T7 promoter, which drives the expression of the gene encoding the target protein [3, 5]. The rationale behind BL21(DE3) is very simple: the higher the mRNA levels, the more protein can be produced. Notably, P*lac*UV5 is in BL21(DE3) a poorly-titratable promoter [7–9]. Expression of genes encoding target proteins, in particular those encoding membrane and secretory proteins, can be toxic to BL21(DE3) [10–13]. The toxicity of membrane and secretory protein production appears to be mainly caused by saturation of the capacity of the machineries involved in the biogenesis of these proteins [8, 11, 14, 15]. Saturating the capacity of the machineries involved in the biogenesis of membrane and secretory proteins negatively affects both biomass formation and the production yields of the target membrane and secretory proteins, e.g., due to the misfolding and aggregation of proteins in the cytoplasm. [8, 11, 14, 15]. In this respect it should be noted that it is preferred to produce membrane proteins in a membrane system rather than in inclusion bodies to facilitate their isolation for structural and functional studies [16].

To harmonize the expression intensity of a gene encoding a target membrane or secretory protein with the capacity of the biogenesis machinery of the protein, we previously developed the BL21(DE3)-derived Lemo21(DE3) strain [8, 12, 14]. The rationale behind Lemo21(DE3) is that the activity of the T7 RNAP can be modulated by the titratable production of its natural inhibitor, T7 lysozyme. The gene encoding the T7 lysozyme is located on the pLemo plasmid and its expression is under the control of a rhamnose promoter. This promoter is well-titratable, meaning that the amount of rhamnose added to a culture correlates with the amount of protein produced [17]. Lemo21(DE3) has been widely and successfully used to identify the best target gene expression intensity for the optimal T7-based production of a variety of proteins (e.g., [8, 12, 14, 18–24]). A disadvantage of the Lemo21(DE3) setup is that the system is based on two plasmids, a T7-based pET-expression vector and pLemo. Hence, the Lemo21(DE3) setup requires two antibiotic markers and complicates the use of helper plasmids. Therefore, it would be desirable to simplify the Lemo21(DE3) setup by combining the key elements of a T7-based expression vector and pLemo in one vector.

In this study we describe the construction and validation of the pReX vector, which is a simplified and more versatile alternative for the widely used Lemo21(DE3)-setup. The pReX vector was constructed by incorporating the part of pLemo encoding the T7 lysozyme under control of the rhamnose promoter in a standard T7-based expression vector. We show that pReX is easy to use and performs in a similar manner as Lemo21(DE3) for optimizing the production of membrane and secretory proteins. Finally, we show that pReX has the additional advantage that it greatly facilitates the use of a helper plasmid.

## Results

### Construction of pReX, a T7-based expression vector enabling regulated target gene expression

**Fig. 1:**
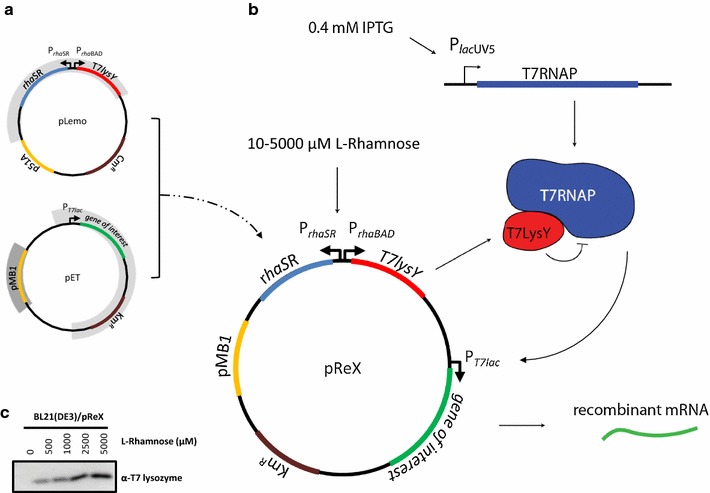
Construction of pReX. **a** The Lemo21(DE3) setup is based on two plasmids; a T7-based pET-expression vector and pLemo. The T7-based pET-expression vector, which has a T7 promoter that contains a *lac*-operator site (T7*lac*), a kanamycin resistance marker (Km-^R^) and a pMB1 origin of replication, is used for the expression of the gene encoding the target protein (gene of interest). pLemo is used for the l-rhamnose inducible P*rhaBAD* promoter-based expression of the gene encoding the T7 lysozyme (*T7lys*). In addition, pLemo contains the *rhaSR* genes encoding the regulatory proteins RhaS and RhaR, a p15A origin of replication and a chloramphenicol (Cm^R^) resistance marker. **b** pReX was created by incorporating the part of pLemo containing the gene encoding the T7 lysozyme along with the l-rhamnose inducible P*rhaBAD* promoter governing its expression and the *rhaSR* genes in a pET-vector with a kanamycin resistance marker (Km^R^) (details of the construction of pReX can be found in the “Methods” section). The idea behind pReX is that it can be used to modulate the activity of the T7 RNA Polymerase (T7 RNAP) in BL21(DE3) and derivatives thereof by adding different amounts of l-rhamnose to the culture medium, which results in the controlled production of the T7 RNAP inhibitor T7 Lysozyme (T7LysY). Notably, the IPTG inducible promoter (P*lac*UV5) governing the expression of the gene encoding the T7 RNAP in BL21(DE3) is only poorly titratable. **c** pReX was transformed into *E. coli* BL21(DE3), and the expression of the gene encoding T7 lysozyme was induced for 4 h with varying l-rhamnose concentrations (0, 500, 1000, 2500 and 5000 µM). Subsequently, T7 lysozyme accumulation levels in whole cell lysates representing equal amounts of cells were monitored by immuno-blotting

The aim of this study was to simplify the two-plasmid based setup of the Lemo21(DE3) system by creating a T7-based expression vector that enables regulated gene expression (Fig. 1a). To this end, we incorporated the part of pLemo encoding the gene for the T7 lysozyme along with the L-rhamnose inducible *rhaBAD* promoter (P*rhaBAD*) governing its expression, in a standard T7-based pET-expression vector. This resulted in the pReX expression vector (ReX stands for Regulated gene eXpression) (Fig. 1b). Next, pReX was transformed into *E. coli* BL21(DE3), and the expression of the gene encoding T7 lysozyme was induced for 4 h with varying l-rhamnose concentrations (0, 500, 1000, 2500 and 5000 µM). Subsequently, T7 lysozyme accumulation levels in whole cell lysates were monitored by immuno-blotting (Fig. 1c). There is a clear relationship between the amount of l-rhamnose added to the culture and the T7 lysozyme present in cells. This indicates that pReX can mediate the titratable production of the T7 lysozyme. Therefore, as a next step, we evaluated the production of two model membrane proteins using the pReX expression vector in BL21(DE3) using Lemo21(DE3) as a reference.

### pReX-based optimization of membrane protein production

The integral membrane chaperone YidC and the glutamate proton symporter GltP were used as model membrane proteins to evaluate the performance of pReX [14]. Both YidC and GltP have successfully been used in the past to evaluate *E. coli*-based protein production systems [13, 14]. As mentioned in the “Background” section, it is preferred to produce membrane proteins in a membrane system rather than in inclusion bodies, since it greatly facilitates their isolation for structural and functional studies [16]. Therefore, to facilitate the detection of in the cytoplasmic membrane produced YidC and GltP, both membrane proteins were C-terminally fused to GFP [25]. The GFP moiety only folds properly and becomes fluorescent when the membrane protein-GFP fusion is inserted in the cytoplasmic membrane [25–27]. When the membrane protein-GFP fusion aggregates in the cytoplasm the GFP moiety does not fold properly and does not fluoresce.

**Fig. 2:**
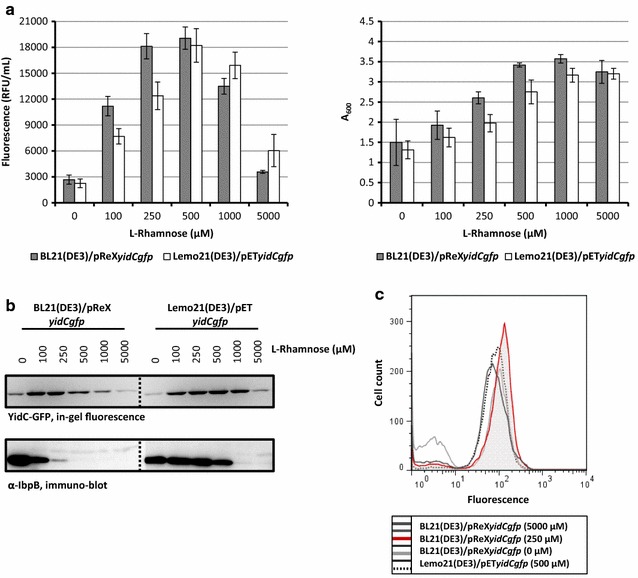
Optimizing the production of the membrane protein YidC using pReX. BL21(DE3) cells harboring pReX*yidCgfp* and Lemo21(DE3) cells harboring pET*yidCgfp* were cultured in LB medium at 30 °C at varying concentrations of l-rhamnose as indicated. All measurements were done 8 h after the addition of IPTG to induce the expression of *t7rnap*. **a** Left panel: to assess the effect of different l-rhamnose concentrations on the production of YidC-GFP in the cytoplasmic membrane, we monitored the fluorescence (relative fluorescence unit, RFU) per milliliter of culture. Right panel: the effect of YidC–GFP production at different l-rhamnose concentrations on biomass formation was monitored by measuring the A_600_. The production of YidC-GFP under Lemo21(DE3) and pReX-based optimal conditions corresponds to 14.0 and 14.7 mg of protein produced per liter of culture, respectively [25]. **b** Top panel: the integrity of the YidC–GFP fusion produced in the cytoplasmic membrane at different l-rhamnose concentrations was monitored using in-gel fluorescence. Bottom panel: accumulation levels of IbpB in cells producing YidC–GFP at different l-rhamnose concentrations were monitored by immuno-blotting using an antibody against IbpB. **c** Using flow cytometry the amount of YidC-GFP fusion produced in the cytoplasmic membrane per cell was assessed. Traces of BL21(DE3)/pReX*yidCgfp* and Lemo21(DE3)/pET*yidCgfp* cells cultured at the optimal l-rhamnose concentration (maximal amount of fluorescent protein per milliliter of culture) are in red. Traces of the same cells cultured in the absence of l-rhamnose (control) are in gray

First, YidC-GFP was produced using the BL21(DE3)/pReX and Lemo21(DE3)/pET setups at varying l-rhamnose concentrations. Both biomass formation (A_600_) and fluorescence (GFP fluorescence per ml of culture) in BL21(DE3)/pReX and Lemo21(DE3)/pET-based cultures were monitored (Fig. 2a). The optimum l-rhamnose concentration for the BL21(DE3)/pReX-based production of YidC-GFP was slightly lower than the one found when the Lemo21(DE3)/pET setup was used to produce YidC-GFP. Using in-gel fluorescence it was shown that the full length YidC-GFP fusion was produced (Fig. 2b, upper panel).

It has been shown that the saturation of the capacity of the membrane protein biogenesis machinery leads to the misfolding/aggregation of proteins in the cytoplasm [8, 11, 14]. The misfolding/aggregation of proteins in the cytoplasm induces the expression of the gene encoding inclusion body protein B (IbpB) [28, 29]. Therefore, to monitor if the production of YidC driven by different target gene expression intensities leads to protein misfolding/aggregation in the cytoplasm, levels of IbpB in cells cultured at different l-rhamnose concentrations were monitored using immuno-blotting (Fig.  2b, lower panel) [28, 29]. At lower l-rhamnose concentrations, i.e., at higher target gene expression intensities, cells contained significant levels of IbpB. In contrast, at higher l-rhamnose concentrations, i.e., at lower target gene expression intensities, IbpB levels were reduced. This shows that there is a protein accumulation/folding problem in the cytoplasm if the expression level of the gene encoding YidC-GFP is too high, which is due to saturation of the capacity of the membrane protein biogenesis machinery [8, 11]. Using flow cytometry, GFP fluorescence in individual cells cultured at the optimal l-rhamnose concentration was monitored (Fig. 2c). Both the BL21(DE3)/pReX and Lemo21(DE3)/pET-based cultures producing YidC-GFP at the optimal L-rhamnose concentration consisted of a homogenous population of cells [14]. This indicates that at these l-rhamnose concentrations target gene expression intensity is balanced with the capacity of the membrane protein biogenesis machinery.

**Fig. 3:**
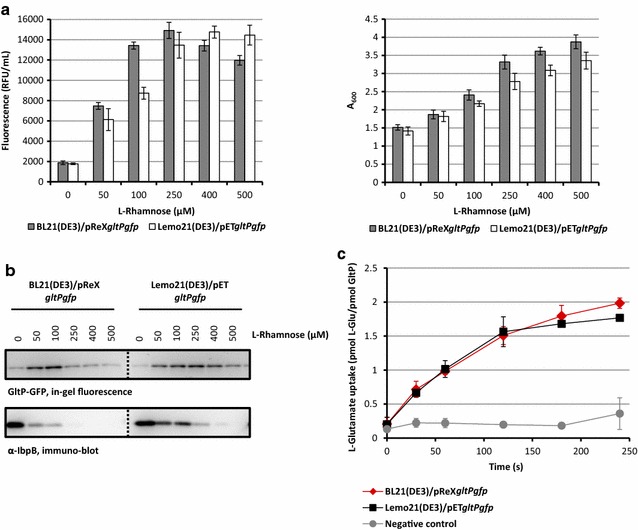
Optimizing the production of the membrane protein GltP using pReX. BL21(DE3) cells harbouring pReX*gltPgfp* and Lemo21(DE3) cells harboring pET*gltPgfp* were cultured and target gene expression was induced as described in Fig. 2. **a** Left panel: to assess the effect of different l-rhamnose concentrations on GltP–GFP production levels, we monitored fluorescence (relative fluorescence unit, RFU) per milliliter of culture. Right panel. The effect of GltP-GFP production in the absence and presence of increasing concentrations of l-rhamnose on biomass formation was monitored by measuring the A_600_. The production of GltP-GFP under Lemo21(DE3) and pReX-based optimal conditions corresponds to 9.6 and 9.7 mg of protein produced per liter of culture, respectively [25]. **b** Top panel: the integrity of the GltP–GFP fusion produced at different l-rhamnose concentrations was monitored using in-gel fluorescence. Bottom panel. Accumulation levels of IbpB in cells producing GltP–GFP at different l-rhamnose concentrations were monitored by immuno-blotting using an antibody against IbpB. **c** GltP-GFP was purified from the membranes of BL21(DE3)/pReX and Lemo21(DE3)/pET cells cultured at the optimal l-rhamnose concentration. Approximately 2.5 mg of GltP-GFP was isolated from 1 l of Lemo21(DE3) and pReX-based cultures. Equal amounts of isolated GltP-GFP were subsequently incorporated in liposomes, and glutamate uptake was determined. Plain liposomes were used as a control

Encouraged by the observation that the optimized BL21(DE3)/pReX-based production of YidC-GFP matches the optimized Lemo21(DE3)/pET-based production of YidC-GFP, we decided to also monitor the production of GltP-GFP using the BL21(DE3)/pReX and Lemo21(DE3)/pET setups at varying l-rhamnose concentrations. Both biomass formation and fluorescence in BL21(DE3)/pReX and Lemo21(DE3)/pET-based cultures followed the same trend (Fig. 3a). The optimal l-rhamnose concentration for the BL21(DE3)/pReX-based production of GltP-GFP was also slightly lower than the one for the Lemo21(DE3)/pET-based production of GltP-GFP. Using in-gel fluorescence it was shown that the full length GltP-GFP fusion was produced (Fig. 3b, upper panel). At lower l-rhamnose concentrations also GltP-GFP producing cells contained significant levels of IbpB and the levels of IbpB decreased with an increase in l-rhamnose concentration in the medium (Fig. 3b, lower panel). Using flow cytometry it was shown that both the BL21(DE3)/pReX and Lemo21(DE3)/pET-based cultures producing GltP-GFP at the optimal L-rhamnose concentration consisted of a homogenous population of cells. (Additional file 1: Figure S1A). Next, to assess the quality of GltP-GFP produced under optimal conditions using the BL21(DE3)/pReX and Lemo21(DE3)/pET setups, the protein was isolated and subsequently reconstituted into liposomes so that glutamate transport activity could be monitored (Fig. 3c) [13, 14, 30]. The glutamate uptake experiments show that GltP-GFP produced using the BL21(DE3)/pReX and Lemo21(DE3)/pET setups is capable of transporting glutamate equally well.

Taken together, the membrane proteins YidC and GltP could both be readily produced using the pReX expression vector in combination with BL21(DE3), and to levels that are similar to the ones when the Lemo21(DE3)/pET-based setup was used for the production of the two proteins. This prompted us to also evaluate the use of pReX for the production of a secretory protein.

### pReX-based optimization of protein production in the periplasm

In *E. coli*, soluble heterologous proteins can be produced in the cytoplasm as well as in the periplasm. There are two important reasons to produce soluble heterologous proteins, in particular ones containing disulfide bonds, in the periplasm rather than the cytoplasm. Firstly, it is easier to isolate a protein from the periplasm than from whole cell lysates. Secondly and more importantly, in the oxidizing environment of the periplasm the disulfide bond formation (Dsb)-system catalyzes the formation of disulfide bonds [31, 32]. However, it has been shown that the production of secretory proteins in the periplasm can also be hampered by saturating the machinery involved in the biogenesis of these proteins [12]. Here, to evaluate the use of pReX to optimize the production of a protein in the periplasm we used as a model protein superfolder GFP N-terminally fused to a modified DsbA signal sequence (DsbA*SfGFP) [12, 13, 33].

**Fig. 4:**
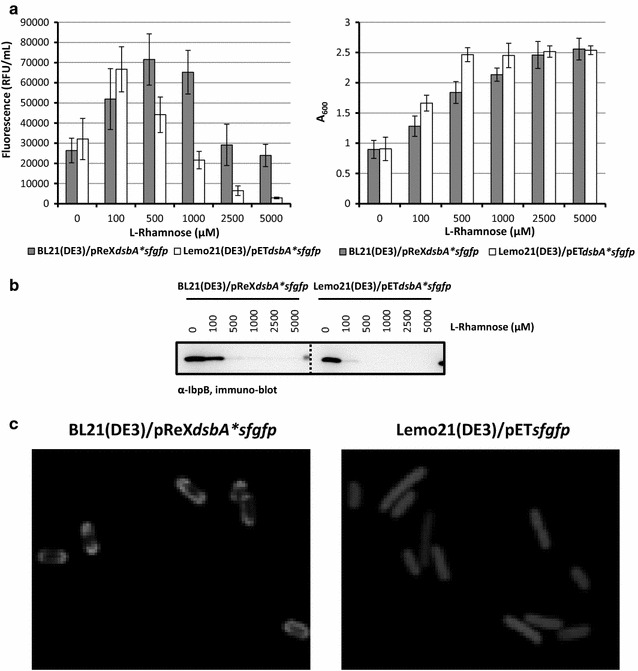
pReX based optimization of the production of secreted SfGFP. BL21(DE3) cells harbouring pReXdsbA*sfgfp and Lemo21(DE3) cells harboring pETdsbA*sfgfp were cultured in LB medium at 30 °C. The expression of the gene encoding secretory SfGFP was induced with 400 μM IPTG for 4 h. l-rhamnose was present as indicated. **a** Left panel: the effect of the production of secreted SfGFP following varying gene expression levels on protein yields was monitored as fluorescence per ml of culture. The production of SfGFP under Lemo21(DE3) and pReX-based optimal conditions corresponds to 17.1 mg and 18.3 of protein produced per liter of culture [25]. Right panel The effect of the production of secretory SfGFP following varying gene expression levels on biomass formation was monitored by measuring the A_600_. **b** Accumulation levels of IbpB in cells producing secretory SfGFP at varying l-rhamnose concentrations were monitored by immuno-blotting using an antibody against IbpB. **c** The localization of secretory SfGFP in BL21(DE3)/pReX and Lemo21(DE3)/pET cells cultured in the presence of the optimal concentration of l-rhamnose was monitored directly in whole cells using fluorescence microscopy. As a control Lemo21(DE3)/pET*sfgfp* cells producing cytoplasmic SfGFP (i.e., SfGFP not equipped with a signal sequence) were included (a version of the pictures in colour can be found in Additional file 1: Figure S2)

Secretory SfGFP was produced using the BL21(DE3)/pReX and Lemo21(DE3)/pET setups at varying l-rhamnose concentrations, and biomass formation (A_600_) and fluorescence in BL21(DE3)/pReX and Lemo21(DE3)/pET-based cultures was monitored (Fig. 4a). Now the optimal l-rhamnose concentration for the BL21(DE3)/pReX-based production of the target protein was slightly higher than the one for the optimal Lemo21(DE3)/pET-based production of secretory SfGFP. To monitor if the production of secretory SfGFP can lead to protein misfolding/aggregation in the cytoplasm, levels of inclusion body protein B (IbpB) were monitored using immuno-blotting (Fig. 4b). At lower l-rhamnose concentrations, i.e., at higher target gene expression intensities, cells contained significant levels of IbpB. In contrast, at higher l-rhamnose concentrations, i.e., at lower target gene expression intensities, IbpB levels were reduced. This indicates that there is a protein accumulation/folding problem in the cytoplasm when the expression intensity of the gene encoding secretory SfGFP is too high, which is likely due to saturation of the capacity of the machinery involved in the biogenesis of secretory proteins [12]. Analysis of BL21(DE3)/pReX and Lemo21(DE3)/pET cells producing secretory SfGFP cultured at the optimal l-rhamnose concentration using fluorescence microscopy revealed a halo of fluorescence (Fig. 4c). This observation indicates that secretory SfGFP, produced by BL21(DE3)/pReX and Lemo21(DE3)/pET cells at the optimal l-rhamnose concentration is indeed directed to the periplasm [12, 13]. Using flow cytometry, it was shown that cultures producing secreted SfGFP at the optimal L-rhamnose concentration consisted of a homogenous population of cells (Additional file 1: Figure S1B). This indicates that the capacity of the machinery involved in the biogenesis of secretory proteins is not saturated under these conditions, which is in keeping with the observation that IbpB cannot be detected. Taken together, pReX can also be used for optimizing the production of a protein in the periplasm.

### pReX facilitates the use of a helper plasmid

The Lemo21(DE3) setup is based on two plasmids; pLemo, which has a p15A origin of replication, and a pET-based vector, which has a pMB1 origin of replication (Fig. 1a). The two plasmid-based setup of Lemo21(DE3) severely complicates the use of an additional plasmid, since the origins of replication of all the three plasmids should be compatible and three different antibiotic resistance markers have to be used. The pReX vector combines the key elements of a pET-vector and pLemo in one vector, with a pMB1 origin of replication (Fig. 1b). Therefore, pReX facilitates the use of helper plasmids required for the efficient T7-based production of proteins.

The production of *c*-type cytochromes in the periplasm of *E. coli,* cultured under aerobic conditions, requires co-expression of the *ccmABCDEFGH* operon [34, 35]. This operon encodes the *E. coli* cytochrome *c* maturation system, which is required for the proper insertion of heme in *c*-type cytochromes [36]. The *ccmABCDEFGH* co-expression vector pEC86 has successfully been used for the production of *c*-type cytochromes in *E. coli* [34, 35]. The p15A-based pEC86 co-expression vector is not compatible with the Lemo21(DE3) setup, since pLemo also has a p15A origin of replication and they both have a chloramphenicol resistance marker. However, pEC86 is compatible with pReX. Here, we used a bacterial octaheme *c* type cytochrome (OCC), N-terminally fused to a single Strep-tag, as a model protein to evaluate the use of a helper plasmid required for the production of a target protein in combination with pReX [13]. The Strep-tag-OCC fusion was N-terminally fused to the OmpA signal sequence to secrete the protein into the periplasm [13]. Hereafter, for reasons of simplicity we refer to the OmpA-Strep-tag-OCC fusion simply as OCC.

**Fig. 5:**
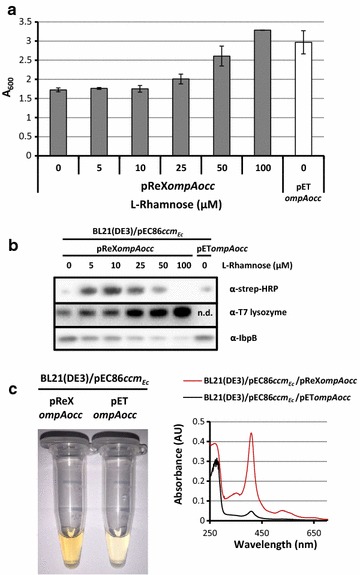
pReX enables the use of a helper plasmid for the efficient production of a cytochrome *c* (OCC) in the periplasm. BL21(DE3)/pReX*ompAocc* and BL21(DE3)/pET*ompAocc* cells also harboring pEC86 were cultured in LB medium at 30 °C. The expression of the gene encoding secretory OCC was induced with 400 μM IPTG for 24 h. l-rhamnose was present as indicated. **a** The effect of the production of secretory OCC following varying gene expression levels on biomass formation was monitored by measuring the A_600_. **b** OCC production in equal amounts of cells isolated from the cultures described in A was monitored using immuno-blotting with an antibody against the Strep-tag. Accumulation levels of the T7 lysozyme, when appropriate, and IbpB in cells producing OCC were monitored by immuno-blotting using antibodies against T7 lysozyme and IbpB, respectively. **c** The OCC was isolated using Strep-tag-based purification from BL21(DE3)/pReX/pEC86 cells cultured at the optimal production condition and BL21(DE3)/pET/pEC86 cells from 3 L cultures as described in the “Methods” section. Left panel: the OCC isolation from BL21(DE3)/pReX/pEC86 cells cultured at the optimal l-rhamnose (the tube on the left) resulted in material with a distinct red color under ambient light, which is indicative for mature cytochrome *c*. 0.24 mg of protein was isolated from 1 l of culture. The colour of the material isolated from BL21(DE3)/pET/pEC86 cells (the tube on the left) was much lighter. 0.04 mg of protein was isolated from 1 l of culture. Right panel: optical spectra of the material shown in the left panel recorded corroborated that much more mature OCC was produced using the BL21(DE3)/pReX/pEC86 setup compared to the BL21(DE3)/pReX/pEC86 setup

The gene encoding the secretory OCC was expressed from the pReX vector and a standard pET vector in BL21(DE3) also harboring the *ccmABCDEFGH* co-expression vector pEC86. Using the BL21(DE3)/pReX setup the OCC was produced in the presence of varying concentrations of l-rhamnose. In both BL21(DE3)/pReX and BL21(DE3)/pET-based cultures, biomass formation (A_600_) and OCC production using immuno-blotting with an antibody against the Strep-tag, were monitored (Fig. 5a, b). Varying the l-rhamnose concentrations in the medium of BL21(DE3)/pReX-based cultures had a clear effect on biomass formation and OCC accumulation levels; 10 μM rhamnose appeared the optimum concentration of l-rhamnose for the production of the OCC. The production of the OCC using the optimal pReX-based setup was significantly better than the production of the OCC using a standard pET vector using BL21(DE3) containing pEC86. As for the other targets, we also monitored the accumulation levels of IbpB in whole cell lysates using immuno-blotting (Fig. 5b). The immuno-blot indicated that the optimal pReX-based condition for the production of OCC was accompanied by only mild stress, whereas the pReX-based condition with 0 μM l-rhamnose and the pET-based condition were accompanied by considerable stress.

The OCC from a pReX-based culture supplemented with the optimal l-rhamnose concentration (10 μM) and the OCC from a pET-based culture were isolated using the Strep-tag (Fig. 5c). The color and the optical spectrum of the OCC isolated from BL21(DE3)/pReX and BL21(DE3)/pET-based cultures indicated that a larger amount of mature cytochrome was produced using the BL21(DE3)/pReX setup rather than the BL21(DE3)/pET setup.

Our combined observations indicate that the bands that are detected in the immuno-blotting experiment shown in Fig. 5b indeed represent the mature, i.e., the periplasmic, heme binding form of OCC. This implies that the not properly targeted precursor form of OCC, although it induces protein misfolding/aggregation stress in the cytoplasm, is at least partially degraded so that it cannot be detected with an antibody recognizing the Strep-tag.

Taken together, using the production of a bacterial octaheme *c*-type cytochrome as an example, it was shown that pReX can facilitate the use of a helper plasmid required for protein production.

### Concluding remarks

Aim of the study was to simplify the Lemo21(DE3)-setup. Therefore, we have created the pReX expression vector for optimizing the T7-based production of proteins. Besides all elements required for the T7-based production of proteins, pReX also contains the gene encoding the T7 lysozyme under control of the titratable rhamnose promoter. Therefore, pReX enables regulated T7-based target gene expression by varying the l-rhamnose concentration in the medium. Here, we have shown that by using pReX, the production of both membrane and secretory proteins can be readily optimized. It is of note that the use of pReX is not restricted to optimizing the production of membrane and secretory proteins. Importantly, as shown in this report, pReX greatly facilitates the use of helper plasmids for e.g., the co-production of chaperones, and it can in principle be used in any T7 RNAP-based strain. Thus, pReX is a versatile tool for the T7-based production of challenging proteins.

## Methods

### Strains and plasmids

In this study, the *E. coli* BL21(DE3) and the from BL21(DE3)-derived Lemo21(DE3) protein production strains were used [5, 8]. Lemo21(DE3) is BL21(DE3) harboring a pACYC-derived vector containing the gene encoding the T7 lysozyme under the control of the l-rhamnose inducible P*rhaBAD* promoter. Notably, the T7 lysozyme K128Y variant that has no amidase activity but retains full inhibition of T7 RNA polymerase was used [8, 37]. In BL21(DE3), the genes encoding the target proteins GltP-GFP, YidC-GFP, DsbA*SfGFP and OmpA-Strep-tag-OCC were expressed from pReX (see below). The gene encoding OmpA-Strep-tag-OCC was also expressed in BL21(DE3) from a modified pET22a vector [13]. The *ccmABCDEFGH* co-expression vector pEC86 was used to facilitate the production of *c*-type cytochromes in the periplasm of BL21(DE3) [34, 35]. In Lemo21(DE3) the genes encoding the target proteins GltP-GFP, YidC-GFP and DsbA*SfGFP were expressed from a pET28a + -derived vector as described before [12, 14].

### Culture media and expression conditions

Cells were grown aerobically at 30 °C and 200 rpm in 24-well plates (unless stated otherwise), in Lysogeny broth (LB) medium (Difco). This setup was used throughout this study since for the Lemo21(DE3)-based production it gives on average the best results and it is easy to implement by any laboratory [12, 14]. If required, cultures were supplemented with 50 μg/ml kanamycin (for pReX and its derivatives, and all pET-based vectors, except for the one used to produce OCC), 30 μg/ml chloramphenicol (for pLemo and pEC86) and 100 μg/ml ampicillin (for the pET-based vector used to produce OmpA-Strep-tag-OCC). Lemo21(DE3) harboring a pET-based expression vector and BL21(DE3) harboring pReX or a pReX derivative were grown in the absence and presence of increasing concentrations of l-rhamnose as indicated. At an A_600_ of ~ 0.5 target gene expression was induced by adding 400 μM IPTG for periods of times as indicated. Growth was monitored by measuring the A_600_ with an UV-1601 spectrophotometer (Shimadzu). Standard deviations shown in figures of culturing experiments are based on at least three biologically independent experiments.

### Construction of pReX and its derivatives

The part of the pLemo plasmid comprising the gene encoding the T7 lysozyme under control of the P*rhaBAD* promoter, the *rhaRS* genes encoding the regulatory proteins RhaS and RhaR as well as the p15A origin of replication was PCR amplified using primers P59_EagILemo_fw and P60_KpnIMod_rv (Additional file 1: Table S1)(Fig. 1). Part of the pET28a + -derived vector pMOD comprising the multiple cloning site (MCS) and the gene encoding the kanamycin marker (Km^R^) was PCR amplified using primers P57_KpnIMod_fw and P58_EagIMod_rv (Additional file 1: Table S1) [13]. The PCR products were purified, digested with *Eag*I and *Kpn*I, and subsequently used to generate pReX-p15A by means of ligation. Next, the p15A origin of replication of pReX-p15A was exchanged for the pMB1 origin of replication using homology cloning. The pMB1 origin of replication of pET28a + was PCR amplified using primers pLemoMOD_pMB1_Ins_fw and –rv (Additional file 1: Table S1). Primers pLemoMOD_Vec_p15A_fw and –rv were used to PCR amplify pReX-p15A without its p15A origin of replication. Using homology cloning the two DNA molecules were combined to generate the pReX plasmid as depicted in Fig. 1 [38, 39]. Genes encoding target proteins (GltP-GFP, YidC-GFP, DsbA-SfGFP, and OmpA-OCC) were inserted in the MCS of pReX by means of homology cloning, using the pET-T7-Vec fw and pET-Vec-rv primers to amplify the plasmid backbone and pET-T7-Ins-fw and pET-Ins-rv primers to amplify the target gene from previously described pT7-based expression vectors (Additional file 1: Table S1) [12–14, 38, 39]. The sequences of all the primers used for engineering pReX, and for inserting the genes encoding the various target proteins in this vector are listed in Additional file 1: Table S1.

### Whole cell fluorescence measurements and flow cytometry

Production of membrane protein GFP fusions and secretory SfGFP were monitored using whole-cell fluorescence as described before [25]. Standard deviations are based on a minimum of three biologically independent experiments. GFP fluorescence was analyzed on a single cell level by flow cytometry using a FACSCalibur instrument (BD Biosciences) as described before [13]. FM4-64 membrane staining was used to discriminate between cells and background signal. The FlowJo software (Treestar) was used for raw data analysis/processing.

### SDS-PAGE, in-gel fluorescence and immuno-blotting

Whole cell lysates (0.05 A_600_ units) were analyzed by standard SDS-PAGE using standard polyacrylamide gels followed by either in-gel fluorescence or immuno-blotting as described before [13]. His-tagged target membrane proteins were detected using an HRP-conjugated α-His antibody (ThermoFisher) recognizing the C-terminal His-tag. OCC was detected using an HRP-conjugated α-Strep antibody (IBA life sciences) [13]. Both T7 lysozyme and IbpB levels were monitored using antisera from our sera collection, followed by incubation with a secondary HRP-conjugated goat-α-rabbit antibody (Bio-Rad). Proteins were visualized using the ECL-system (GE Healthcare) according to the instructions of the manufacturer and a Fuji LAS-1000 charge coupled device (CCD) camera.

### Glutamate transport assay

Purification of GltP-GFP, reconstitution into proteoliposomes and [^14^C]glutamate transport assays were performed as described before [13].

### Fluorescence microscopy

Prior to microscopy, cells were fixed using cross-linking reagents. Cells corresponding to 1 A_600_ unit were harvested (4000 × g, 2 min) and resuspended in 1 ml phosphate buffered saline (PBS) pH 7.4. Subsequently, 1 ml fixing solution (5.6% Formaldehyde, 0.08% Glutaraldehyde in PBS) was added and cells were incubated for 15 min at room temperature. Subsequently, cells were washed three times with PBS and resuspended in 100 μl PBS. 2 μl of the cell suspension was mounted on a glass slide. Fluorescence images of cells producing secretory SfGFP or as a control cytoplasmic SfGFP were obtained using a light scanning microscope (LSM 700) set-up (Zeiss). The resulting images were processed with the AxioVision 4.5 software (Zeiss).

### Isolation of OCC

Cells from 3 L of BL21(DE3)/pReX*ompAStrepocc*/pEC86 and BL21(DE3)/pET22*ompAStrepocc*/pEC86 cultures, with the optimal l-rhamnose concentration added and induced at an A_600_ of ~ 0.5 with 400 µM IPTG for 24 h were harvested by centrifugation (5000 × *g*, 15 min, 4 °C). The cell pellet was snap-frozen in liquid nitrogen. All subsequent steps were carried out either on ice or at 4 °C. The snap-frozen cell pellet was thawed on ice and subsequently resuspended under gentle agitation in 1 ml ice-cold isolation buffer (50 mM Tris–HCl pH 7.5, 700 mM NaCl, 2 mM MgCl_2_) per 120 A_600_ units of cells, supplemented with 0.5 mg/ml Pefablock. Subsequently, cells were broken with five passes through an Emulsiflex-C3 (Avestin), at 10,000–15,000 psi. The lysate was cleared of unbroken cells by centrifugation (8000 ×*g*, 3 × 20 min, 4 °C). OCC was isolated from the cleared lysate using a Streptavidin column (IBA Biosciences). The column was equilibrated with 5 column volumes of binding buffer (50 mM Tris–HCl pH 7.5, 300 mM NaCl). After loading OCC onto the column, it was eluted with elution buffer (50 mM Tris–HCl pH 7.5, 300 mM NaCl, 5 mM Desthiobiotin). Eluted fractions were analyzed by SDS-PAGE followed by immuno-blotting. Protein concentrations were determined using the BCA assay (ThermoFisher). Optical spectra were recorded with a UV-1800 UV–Vis spectrophotometer (Shimadzu), as described previously [40].

## Additional file

**Additional file 1.** In the supplemental material section primers used in this study are provided and results from the membrane and secretory protein production experiments are presented.

## Abbreviations

GFP: green fluorescent protein
IbpB: inclusion body protein B
IPTG: isopropyl-β-d-1-thiogalactopyranoside
OCC: octaheme *c*-type cytochrome
PBS: phosphate buffered saline
MCS: multiple cloning site
RFU: relative fluorescence unit
SfGFP: superfolder green fluorescent protein
T7 lys: T7 lysozyme
T7 RNAP: T7 RNA polymerase
