# Supplementary material for: The tunable pReX expression vector enables optimizing the T7-based production of membrane and secretory proteins in E. coli
Source: Microb Cell Fact. 2017 Dec 16;16:226. doi: 10.1186/s12934-017-0840-4 (PMC5732464; doi:10.1186/s12934-017-0840-4)

Supplementary Table 1: The primers used in this study.

Primer name	Primer sequence	Primer description
P57_KpnIMod_fw	5'- ATATATGGTACCTCGCCGAAAATGACCCAG-3'	Forward primer to introduce a *Kpn*I restriction site in the pMOD fragment of pReX
P58_EagIMod_rv	5'- ATATATCGGCCGTCAAGAAGATCCTTTGATCTTTTC-3'	Reverse primer to introduce an *Eag*I restriction site in the pMOD fragment of pReX
P59_EagILemo_fw	5'- ATATATCGGCCGTCACAGGTATTTATTCGGC-3'	Forward primer to introduce an *Eag*I restriction site in the pLemo fragment of pReX
P60_KpnILemo_rv	5'- ATATATGGTACCAGGGCAGGGTCGTTAAATAG-3'	Reverse primer to introduce a *Kpn*I restriction site in the pLemo fragment of pReX
pLemoMOD_pMB1_ins_fw	5'- ACACCCCTTGTATTACTGTTTATG-3'	Forward primer to amplify the pMB1 origin of replication
pLemoMOD_pMB1_ins_rv	5'- CTGACTTCAGGTGCCTGTGGAACACCTACATC-3'	Reverse primer to amplify the pMB1 origin of replication
pLemoMOD_Vec_p15A_fw	5'- CACCTGAAGTCAGCCCC-3'	Forward primer to amplify the vector backbone of pReX
pLemoMOD_Vec_p15A_rv	5'- CATAAACAGTAATACAAGGGGTGT-3'	Reverse primer to amplify the vector backbone of pReX
pET-T7-Ins_fw	5'- ATTAATACGACTCACTATAGGG-3'	Forward primer to subclone target genes into pReX
pET-Ter-Ins_rv	5'- ATCCGGATATAGTTCCTCC-3'	Reverse primer to subclone target genes into pReX
pET-Ter-Vec_fw	5'- GGAGGAACTATATCCGGAT-3'	Forward primer to amplify the vector backbone of pReX
pET-T7-Vec_rv	5'- CCCTATAGTGAGTCGTATTAAT-3'	Reverse primer to amplify the vector backbone of pReX

Supplementary Figure 1: Flow cytometry-based analysis of GltP-GFP and secreted SfGFP production. BL21(DE3) cells harboring a pReX expression vector with either *gltPgfp* or *dsbA**sfgfp* and Lemo21(DE3) cells harboring a pET expression vector with either *gltPgfp* or *dsbA**sfgfp* were cultured in LB medium at 30°C at varying L-rhamnose concentrations. Cells where induced with IPTG as described in the Methods section. **A.** Using flow cytometry the amount of GltP-GFP fusion produced in the cytoplasmic membrane per cell was assessed. Traces of BL21(DE3)/pReX*gltpgfp* and Lemo21(DE3)/pET*gltpgfp* cells cultured at the optimal L-rhamnose concentration (maximal amount of fluorescent protein per milliliter of culture) are in red and black (dotted), respectively. The trace of the BL21(DE3)/pReX*gltpgfp* cells cultured in the absence of L-rhamnose (0 μM L-rhamnose; control) is in light gray. The trace of the BL21(DE3)/pReX*gltpgfp* cells cultured in the presence of a suboptimal concentration of L-rhamnose (500 μM L-rhamnose; control) is in dark gray. Cells were isolated 8 h after the addition of IPTG. **B.** Using flow cytometry the amount of SfGFP produced in the periplasm per cell was assessed. Traces of BL21(DE3)/pReX*dsbA**sfgfp* and Lemo21(DE3)/pET*dsbA**sfgfp* cells cultured at the optimal L-rhamnose concentration (maximal amount of fluorescent protein per milliliter of culture) are in red and black (dotted), respectively. The trace of the BL21(DE3)/pReX*dsbA**sfgfp* cells cultured in the absence of L-rhamnose (0 μM L-rhamnose; control) is in light gray. The trace of the BL21(DE3)/pReX*gltpgfp* cells cultured in the presence of a suboptimal concentration of L-rhamnose (5000 μM L-rhamnose; control) is in dark gray. The trace of the Lemo21(DE3)/pET*dsbA**sfgfp* cells cultured in the absence of L-rhamnose (0 μM L-rhamnose; control) is in blue. Cells were isolated 4 h after the addition of IPTG.

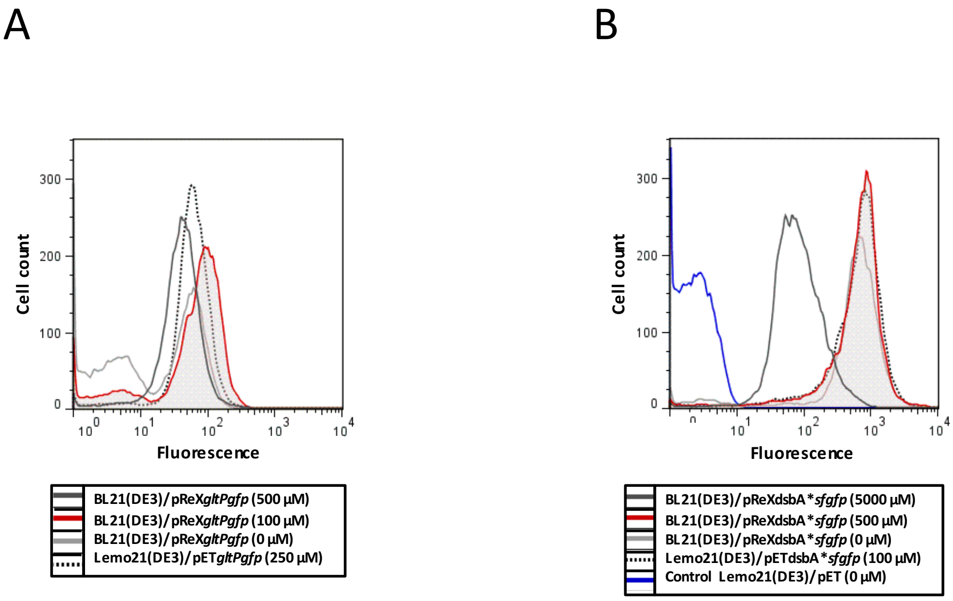
A
B
Cell count
300
200
100
0
Fluorescence
BL21(DE3)/pReXgltPgfp (500 µM)
BL21(DE3)/pReXgltPgfp (100 µM)
BL21(DE3)/pReXgltPgfp (0 µM)
Lemo21(DE3)/pETgltPgfp (250 µM)
BL21(DE3)/pReXdsbA*sfgfp (5000 µM)
BL21(DE3)/pReXdsbA*sfgfp (500 µM)
BL21(DE3)/pReXdsbA*sfgfp (0 µM)
Lemo21(DE3)/pETdsbA*sfgfp (100 µM)
Control Lemo21(DE3)/pET (0 µM)

Supplementary Figure 2: Color version of Figure 4C.

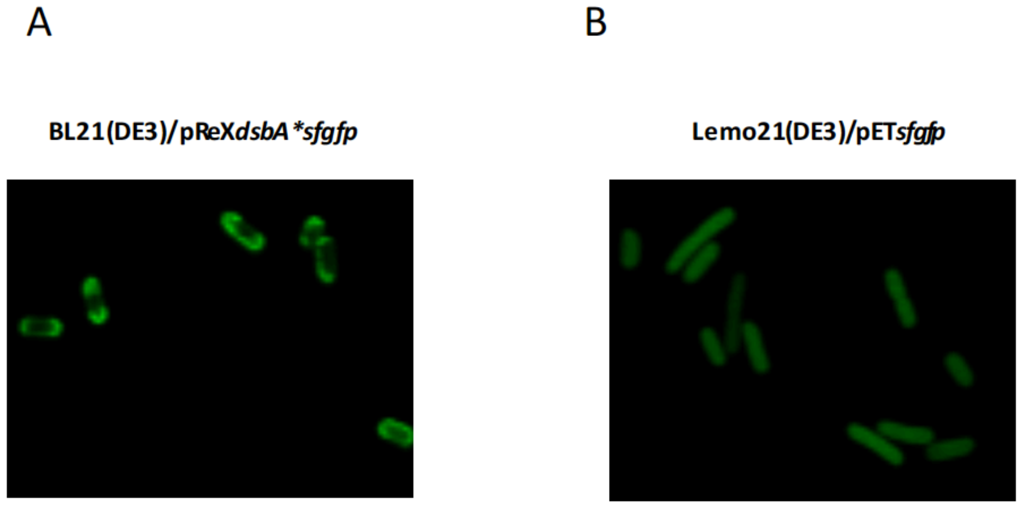

Supplement: Supplementary file 1 — Additional file 1. In the supplemental material section primers used in this study are provided and results from the membrane and secretory protein production experiments are presented. [file 12934_2017_840_MOESM1_ESM.pdf]
